# Evaluation of Salivary and Serum BAFF and TWEAK Levels in Patients with Thalassemia Major and Gingival Inflammation: An Observational Case–Control Study

**DOI:** 10.1055/s-0045-1810423

**Published:** 2025-08-18

**Authors:** Rua'a Riyadh, Maha Sh. Mahmood

**Affiliations:** 1 Department of Periodontics, College of Dentistry, University of Baghdad, Baghdad, Iraq

**Keywords:** thalassemia major, gingival inflammation, BAFF, TWEAK

## Abstract

**Objective:**

Limited research has been conducted to evaluate the relationship between periodontal parameters, systemic biomarkers, and thalassemia major (TM) concerning gingival inflammation. This study aims to assess the potential association of B cell activating factor (BAFF) and tumor necrosis factor weak inducer of apoptosis (TWEAK) with periodontal parameters in patients diagnosed with TM and gingivitis, compared with their healthy counterparts.

**Materials and Methods:**

The current study involved 101 participants aged 18 to 35 years; each subject completed a preliminary assessment to ascertain their eligibility for recruitment. Periodontal parameters were recorded, including the following indices: plaque index, bleeding on probing, and the Oral Hygiene Simplified Index (OHS-I-calculus index). The concentrations of BAFF and TWEAK in saliva and serum were evaluated using enzyme-linked immunosorbent assay.

**Statistical Analysis:**

The Mann–Whitney and the Kruskal–Wallis tests were employed for comparisons, with the Dunn–Bonferroni *post hoc* method applied for pairwise multiple comparisons when applicable. Intraclass correlation coefficient and kappa tests were used to test the reliability of the examiner calibration. Spearman rank correlation was used to assess the relationship between the biomarkers under study and clinical periodontal data. Additionally, multiple linear regression analysis was utilized to investigate the association of biomarkers, TM, and gingivitis while controlling for potential confounding effect of age. *p* -Values below 0.05 were set to be statistically significant.

**Results:**

Both biomarkers were detected in all subjects, with a significant difference in their levels between the groups. Additionally, significant correlations were found between the levels of TWEAK and BAFF and periodontal indices ( *p* < 0.001). Multiple linear regressions demonstrated a significant association between the elevated levels of salivary and serum BAFF and TWEAK with TM and gingival health status ( *p* < 0.05), as for age was not statistically significant.

**Conclusion:**

The findings demonstrate a possible correlation between elevated salivary and serum levels of BAFF and TWEAK and the presence of TM and gingival inflammation, suggesting that these biomarkers may reflect systemic immune alterations in TM in the presence of gingival inflammation.

## Introduction

Thalassemia major (TM) is an autosomal recessive hereditary hematological disease and the most severe form of β-thalassemia. This condition arises from abnormalities in the β chain of the hemoglobin molecule, leading to severe anemia, ineffective erythropoiesis, and a frequent need for regular blood transfusions for patient care. Consequently, individuals suffering from TM often exhibit several immunological disturbances, including functional changes in T-lymphocytes and B-lymphocytes, dysregulation of cytokine production, compromised chemotactic function, and phagocytosis of macrophages and neutrophils. These immunological manifestations are typically secondary effects of blood transfusions and ineffective erythropoiesis. 1 2

Gingival inflammation represents the initial reaction of the periodontium against invading pathogens that can result in more severe periodontal disease. It serves as the body's mechanism to limit the effects of infection and restore tissue integrity. According to studies, gingival inflammation may increase the levels of biomarkers, which can enhance neutrophil and macrophages' phagocytic, secretory, and chemotactic activities. 3 4 5 6 Oral pathogens have a role in periodontal disease, impacting not only the oral cavity but the related systemic diseases such as diabetes mellitus, cardiovascular disease, and rheumatoid arthritis. Both innate and adaptive immune responses may result from chronic gingival inflammation caused by persistent plaque levels (microbial challenges from dysbiotic microbiota). 7 8

B cell activating factor (BAFF), also known as B cell stimulating factor (BLyS), is a member of the tumor necrosis factor (TNF) superfamily and exerts an immune-modulatory effect on both innate and adaptive immune responses. Multiple cell types, including macrophages, B cells, dendritic cells, and T cells, express BAFF; it regulates B lymphocyte proliferation and survival. BAFF expression has been associated with inflammation and infection. 9 TNF-like weak inducer of apoptosis (TWEAK), known as TNFSF12, is classified as a member of the TNF superfamily. It is primarily synthesized by various immune cells, such as neutrophils, activated T cells, natural killer (NK) cells, monocyte\macrophages, plasma cells, and fibroblasts. TWEAK plays a crucial role in regulating multiple biological processes, including cell proliferation, the induction of inflammatory cytokines, angiogenesis, differentiation, and apoptosis. 10 Elevated levels of BAFF and TWEAK have been observed in the oral fluids of patients with periodontitis and gingivitis compared with healthy individuals. These biomarkers have been studied concerning periodontal diseases with other systemic conditions, including rheumatoid arthritis, Sjogren syndrome, osteoporosis, gestational diabetes, and chronic migraine. This suggests a possible association between these cytokines and various systemic diseases. 11 12 13 14 Recent studies have emphasized the potential role of BAFF and TWEAK as salivary biomarkers for periodontal diseases. Yilmaz et al found that salivary BAFF levels were significantly higher in periodontitis patients than in healthy individuals, with a significant reduction observed after periodontal therapy. This indicates that BAFF may be valuable for assessing disease activity and treatment efficacy. A related study by the same authors indicated that elevated baseline levels of TWEAK in gingival tissues were associated with unfavorable healing outcomes over a 12-week follow-up period. This suggests a possible prognostic function of TWEAK in periodontal inflammation and recovery. These results highlight the significance of examining BAFF and TWEAK concerning periodontal changes, including gingivitis, especially in populations with systemic immune dysregulation. 15 16

Research on the relationship between gingival inflammation and TM is limited and occasionally conflicting; this area requires further investigation. The inconclusive findings from previous studies, along with the exclusion of relevant biochemical data in the only systematic review on TM and periodontal health, emphasize the need for more research. 17 Previous studies have focused on the significant changes in the soft tissue profile, including a reduction in tongue size among TM patients compared with healthy controls. 18 Subsequent research has revealed the most common oral manifestations and craniofacial alterations associated with TM. However, these investigations fail to find any association between TM and periodontal disease after assessing certain periodontal indices, such as the plaque index (PI) and bleeding on probing (BOP). 19

Recent research has highlighted a more active role for periodontal resident cells in modulating local immune responses. Dyab et al demonstrated that human gingival fibroblasts express BAFF in response to proinflammatory stimulation, particularly TNF-α, indicating that these cells can directly contribute to B cell survival and function during periodontal inflammation. Immunohistochemical analysis revealed increased BAFF protein expression in both the gingival epithelium and underlying connective tissue in periodontitis lesions, compared with minimal expression in healthy tissue. Moreover, serum BAFF levels were significantly elevated in periodontitis patients. These findings suggest that BAFF not only plays a central role in local periodontal immune activation but may also reflect systemic inflammatory burden. 20

The selection of BAFF and TWEAK as primary inflammatory biomarkers in this study was based on their emerging roles in both systemic immune regulation and local inflammatory responses within periodontal tissues. While BAFF is traditionally associated with B cell activation and has been more extensively studied in the context of periodontitis, 11 limited research has explored its involvement in gingivitis. TWEAK, on the other hand, has not yet been studied in the context of gingivitis, making its inclusion particularly novel. Importantly, BAFF has been reported to be elevated in individuals with TM, suggesting a broader immunopathological role beyond its classical functions. 21 Both BAFF and TWEAK are involved in modulating innate and adaptive immune pathways, including dendritic cell activation, cytokine production, and tissue remodeling processes that are implicated in gingival inflammation. 9 10 11 Investigating these markers in TM patients with gingivitis provides a novel opportunity to explore how systemic immune alterations may influence local inflammatory responses in the periodontium.

The current study hypothesizes that the salivary and serum levels of BAFF and TWEAK biomarkers in patients with TM differ significantly from those in healthy individuals, potentially influenced by the presence or absence of gingival inflammation. This study aims to investigate the salivary and serum levels of BAFF and TWEAK in patients with TM with and without gingival inflammation and to explore their potential role as immunological markers reflecting systemic immune alterations relevant to periodontal health.

## Materials and Methods

### Study Design

This study is an observational case–control investigation conducted from December 2023 to May 2024. Subjects were recruited from patients attending the hereditary blood disorder centers in Baghdad and the Department of Periodontics at the College of Dentistry, University of Baghdad, Iraq. The research adhered to the Declaration of Helsinki regarding human research and complied with ethical standards, obtaining approval from the Ethics Committee at the College of Dentistry, University of Baghdad (Ethical approval number: 853623 on 3–12–2023). This study was assessed and validated following the STROBE (Strengthening the Reporting of Observational Studies in Epidemiology) guideline. Before participation, written informed consent was obtained following a thorough explanation of the study methods to each participant. Participants for each group were selected following a detailed medical and dental history evaluation, with a comprehensive periodontal examination.

### Patient Recruitment and Grouping

The study involved 101 participants aged 18 to 35 years, classified into four distinct groups:

- Group 1: 26 subjects diagnosed with TM and gingivitis (TMG).
- Group 2: 25 subjects with TM and healthy periodontium (TMH).
- Group 3: 25 systemically healthy subjects exhibiting gingivitis (HG).
- Group 4: 25 systemically and periodontally healthy subjects as a control group (HH).

### Eligibility Criteria

The inclusion criteria for selecting patients with TM are as follows:

1. Individuals aged 18 years and older.
2. Must be free from infections, including hepatitis B, hepatitis C, and human immunodeficiency virus.
3. Should be receiving chelation therapy and regular blood transfusions.

Participants in the comparison group must also be free of any systemic diseases.

The exclusion criteria considered the following factors:

1. Presence of systemic diseases other than TM.
2. Status of pregnant or lactating individuals.
3. Use of antimicrobial agents or anti-inflammatory medications within the past 6 months.
4. Smokers.

#### Saliva Collection

Whole unstimulated saliva samples were collected using the passive drooling method before any periodontal examination. Samples were obtained between 8:00 a.m. and 10:00 a.m. to minimize the influence of diurnal variation on the biochemical parameters assessed. Participants were instructed to avoid all oral hygiene practices—including flossing, brushing, mouthwash use, and eating or drinking—for 2 hours before saliva sampling. Participants rinsed their mouths with distilled water for 2 minutes, waited 10 minutes, and then expectorated approximately 3 mL of saliva into sterile tubes, which were subsequently stored at –20°C. 22

#### Serum Sampling

The skin above the antecubital fossa was adequately disinfected before collecting a 3-mL blood sample using venipuncture. Following collection, the blood was centrifuged for 10 minutes at 2,000 revolutions per minute to extract the serum. The serum was preserved at –20°C for future analysis. 23

## Clinical Periodontal Examination

A thorough clinical periodontal evaluation was conducted after collecting saliva and serum samples. This assessment involved measuring the full mouth PI, 24 the Oral Hygiene Simplified Index (OHS-I) for calculus, 25 and determining BOP, 26 which was marked as either positive or negative. A BOP result is considered positive if it occurs within 30 seconds of probing. Clinical examinations were recorded at six sites for each tooth: mesiobuccal, mid-buccal, distobuccal, mesiolingual, mid-lingual, and distolingual. Four surfaces were observed to calculate the PI. The assessment included all teeth except the wisdom teeth and used a calibrated periodontal probe UNC-15 by a single calibrated examiner (R.R.).

According to the clinical criteria defined in the 2017 International World Workshop on the classification of periodontal diseases and disorders, periodontal health diagnosis was based on the absence of clinical attachment loss (CAL), a probing pocket depth (PPD) ≤ 3 mm, and a BOP score less than 10%. As for gingivitis, the absence of CAL, a PPD of 3 mm or less, and a BOP score of more than 30% at all sites. No signs of periodontitis were present in this group of patients. 27

### Inter- and Intraexaminer Calibration

Calibrations between and within the examiners were used to assess the examiner's accuracy and consistency in measuring periodontal parameters, including PL, BOP, and PPD, with the help of a specialist periodontist. For intraexaminer calibration, the researcher took two measurements of five participants separated by 2-hour intervals. To achieve an appropriate degree of agreement, the measures were standardized and aligned. For all clinical parameters, this was demonstrated by an intraclass correlation coefficient above 90% and a kappa test score above 0.75

## Assessment of BAFF and TWEAK Levels

Commercial enzyme-linked immunosorbent assay (ELISA) kits were acquired to quantify the levels of BAFF and TWEAK. The manufacturer's instructions were carefully followed. The microplate included in these kits was precoated with an antibody specific to the cytokines. Samples were introduced into the designated wells, accompanied by a biotin-conjugated antibody specific to BAFF and TWEAK. Avidin conjugated to horseradish peroxidase was subsequently added to each well and incubated. Following this, a tetramethylbenzidine substrate solution was introduced; wells containing cytokines, biotin-conjugated antibodies, and enzyme-conjugated avidin demonstrated a color change. The enzyme-substrate reaction was halted by adding a sulfuric acid solution, and the resulting color change was quantified spectrophotometrically at a wavelength of 450 nm ± 10 nm. The concentrations of the biomarkers in the samples were determined by comparing their optical density to a standard curve. The detection range of the BAFF ELISA kit assay is 62.50 to 4000 pg/mL, and for TWEAK is 7.81 to 500 pg/mL as reported by the manufacturer. All BAFF and TWEAK concentrations measured were within the detectable range of the assay. Both BAFF and TWEAK were detected in all samples and there was no data loss due to degradation or undetected samples.

### Sample Size

The sample size calculation was performed using a pilot study involving the first 10 patients from each group, utilizing the following formula: *n* = ( *r* + 1)/ *r* * *σ* ^2^ ( *Z* 1–β + *Z* 1 *α* /2) ^2^ / *d* ^2^ .

The primary variable of interest was the mean and standard deviation (SD) of BAFF in both the case and control groups. The results indicated that a sample size of 17 patients per group is sufficient. To account for technical dropout, each group was populated with 25 patients. 28

## Statistical Analysis

Statistical analyses were conducted using the Statistical Package for the Social Sciences (SPSS 26). The Shapiro–Wilk test was utilized to evaluate the normality of the distributed data. Results for continuous data were presented as mean ± SD. For comprehensive analysis, the Mann–Whitney test and the Kruskal–Wallis test were employed for comparisons, with the Dunn–Bonferroni *post hoc* method applied for pairwise multiple comparisons when applicable. Intraclass correlation coefficient and kappa tests were used to test the reliability of the examiner calibration. Spearman rank correlation was used to assess the relationship between the biomarkers under study and clinical periodontal data. Additionally, multiple linear regression analysis was utilized to investigate the association of biomarkers with TM, and gingivitis while controlling for potential confounding effect of age differences among groups. *p* -Values below 0.05 were set to be statistically significant.

## Results

### Clinical Analysis

**Fig. 1:**
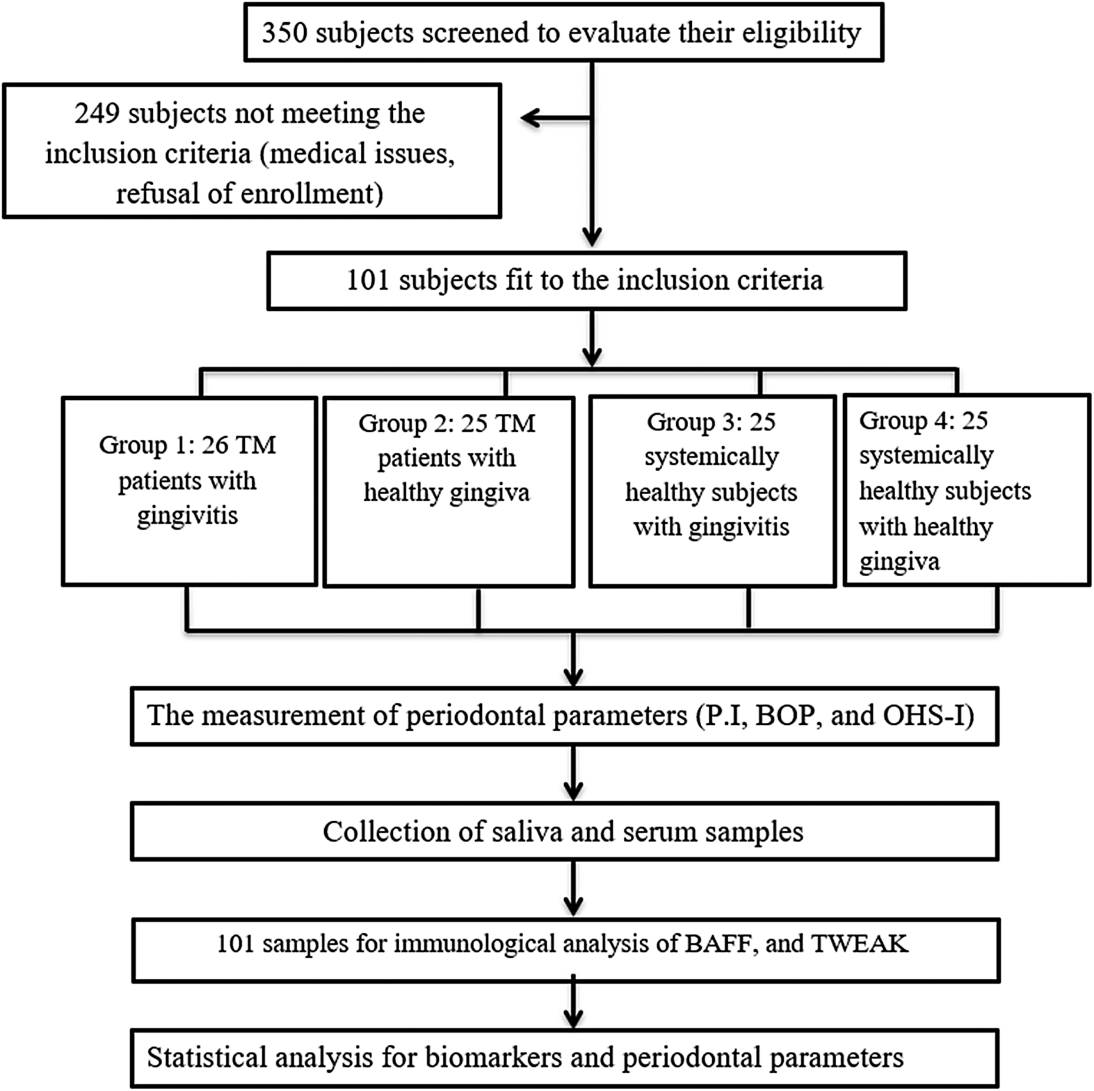
Flowchart illustrating the study design.

The initial assessment included 350 individuals; the application of the eligibility criteria eliminated 249 subjects. Consequently, 101 participants were included in the final analysis, as shown in Fig. 1 . Demographic variables alongside clinical periodontal measurements are presented in Table 1 . Groups 3 and 4 exhibited a statistically significant mean age compared with groups 1 and 2 ( *p* < 0.001). Cross-tabulation disclosed no significant differences between the groups regarding gender. The TMG group and the HG group had significantly higher PI scores compared with the TMG group and HH group ( *p* < 0.001). BOP and OHS-I showed comparable results.

**Table 1:** Demographic and clinical periodontal characteristics

	Thalassemia major		Systemically healthy		*p* -Value
Gingival health	Gingivitis TMG	Healthy TMH	Gingivitis HG	Healthy HH	
Age (mean ± SD)	21 ± 2.62 a b	22.72 ± 4.25 a c	27.08 ± 4.70 c d	25.92 ± 4.18 b d	< 0.001 f
Gender (m/f)	10/16	9/16	13/12	5/20	0.135 e
PI	44.22 ± 10.73 a b	7.69 ± 2.68 a c	34.51 ± 10.03 c d	9.30 ± 2.86 b d	< 0.001 f
BOP	41.18 ± 6.67 a b	7.01 ± 1.42 a c	36.02 ± 4.43 c d	6.22 ± 1.25 b d	< 0.001 f
OHS-I	1.55 ± 0.61 a b	0.12 ± 0.25 a c	1.40 ± 0.56 c d	0.08 ± 0.16 b d	< 0.001 f

Abbreviations: BOP, full-mouth bleeding on probing; OHS-I (calculus), Oral Hygiene Simplified-Index (calculus index); PI, full-mouth plaque index.

Note: Data are reported as mean ± standard deviation. For each parameter, substantial variations across relevant groups are shown by identical superscript low case letters.

a indicates a substantial distinction between group 1 and group 2.

b indicates a substantial distinction between group 1 and group 4.

c indicates a substantial distinction between group 2 and group 3.

d indicates a substantial distinction between group 3 and group 4.

e Groups were compared using the chi-square test.

f Groups were compared using the Kruskal–Wallis test and Mann–Whitney test for pairwise comparison (with Dunn–Bonferroni correction test).

## ELISA Analysis

The circulating levels of the biomarkers under investigation are shown in Table 2 . Differences were statistically significant ( *p* < 0.001) in the mean levels of BAFF and TWEAK in saliva and serum between the TMG and HG groups compared with the TMG and HH groups. The Spearman correlation coefficient analyses were initially conducted using pooled data from all participants ( Table 3 ). To further explore group-specific relationships, Spearman correlation analyses were performed separately within each of the four study groups, as presented in Tables 4 5 6 to 7 . In Table 3 shows that all biomarkers and periodontal parameters are positively correlated in all study participants. Regarding the correlations within each group, in the TMG group, a negative association was found between serum and saliva BAFF ( *p* = 0.014), as illustrated in Table 4 . The positive correlation between saliva BAFF and serum TWEAK and saliva TWEAK was statistically significant ( *p* = 0.024; *p* = 0.048, respectively). Regarding the correlation of periodontal parameters with cytokine levels, both the PI and OHS-I were negatively correlated with saliva BAFF ( *p* = 0.050, *p* = 0.001, respectively) while positively correlated with serum BAFF ( *p* = 0.013, *p* = 0.042, respectively).

**Table 2:** Saliva and serum concentration of BLyS and TWEAK in the study population

	Thalassemia major		Systemically healthy		*p* -Value
Biomarkers	Gingivitis TMG	Healthy TMH	Gingivitis HG	Healthy HH	
Saliva BAFF	1314.794 ± 402.259 a b	590.277 ± 74.255 a c	1023 ± 85.294 c d	570.692 ± 167.487 b d	< 0.001 e
Saliva TWEAK	183.108 ± 38.497 a b	34.196 ± 8.123 a c	112.272 ± 22.211 c d	37.577 ± 7.772 b d	< 0.001 e
Serum BAFF	2164.785 ± 404.202 a b	835.405 ± 96.469 a c	1369.616 ± 254.347 c d	651.435 ± 110.585 b d	< 0.001 e
Serum TWEAK	191.787 ± 49.437 a b	102.396 ± 16.313 a c	168.483 ± 45.323 c d	65.304 ± 19.297 b d	< 0.001 e

Abbreviations: BAFF, B cell activating factor; BLyS, B cell stimulating factor; TWEAK, tumor necrosis factor weak inducer of apoptosis.

Note: All the data are demonstrated as mean ± standard deviation.

For each parameter, substantial variations across relevant groups are shown by identical superscript lower case letters:

a indicates a substantial distinction between group 1 and group 2.

b indicates a substantial distinction between group 1 and group 4.

c indicates a substantial distinction between group 2 and group 3.

d indicates a substantial distinction between group 3 and group 4.

e *p* < 0.01 by the Kruskal–Wallis test and Mann–Whitney test for pairwise comparison (with Dunn–Bonferroni correction test).

**Table 3:** Correlation among BAFF, TWEAK, and clinical periodontal parameters in the study population

		PI	BOP	OHS-I	Saliva BAFF	Saliva TWEAK	Serum BAFF	Serum TWEAK
PI	*R* *p*	1 .						
BOP	*R* *p*	0.869 a 0	1 .					
OHS-I	*R* *p*	0.833 a 0	0.799 a 0	1 .				
Saliva BAFF	*R* *p*	0.738 a 0	0.805 a 0	0.714 a 0	1 .			
Saliva TWEAK	*R* *p*	0.796 a 0	0.790 a 0	0.763 a 0	0.836 a 0	1 .		
Serum BAFF	*R* *p*	0.813 a 0	0.830 a 0	0.793 a 0	0.812 a 0	0.823 a 0	1 .	
Serum TWEAK	*R* *p*	0.680 a 0	0.772 a 0	0.726 a 0	0.768 a 0	0.733 a 0	0.781 a 0	1 .

Abbreviations: BAFF, B cell activating factor; BOP, bleeding on probing; OHS.-I, Oral Hygiene Simplified Index (calculus index); PI, plaque index; *p* , *p* -value; *R* , Spearman correlation coefficient; TWEAK, tumor necrosis factor weak inducer of apoptosis.

a Correlation is significant at the 0.01 level (two-tailed).

**Table 4:** Correlations of salivary and serum biomarkers and periodontal indices in the TM with gingivitis group (TMG)

		PI	BOP	OHS-I	Salivary BAFF	Salivary TWEAK	Serum BAFF	Serum TWEAK
PI	*R* *p*	1.0 00						
BOP	*R* *p*	0.405 0.040	1.000					
OHS-I	*R* *p*	0.338 0.091	−0.020 0.924	1.000				
Salivary BAFF	*R* *p*	**−0.388** ** 0.050 a**	−0.111 0.589	**−0591** **0.001**	1.000			
Salivary TWEAK	*R* *p*	−0.279 0.167	0.066 0.749	−0.066 0.747	**0.392** ** 0.048 a**	1.000		
Serum BAFF	*R* *p*	**0.481** ** 0.013 a**	−0.001 0.995	**0.402** **0.042**	**−0.478** ** 0.014 a**	−0.350 0.080	1.000	
Serum TWEAK	*R* *p*	−0.158 0.440	−0.137 0.504	−0.335 0.094	**0.443** ** 0.024 a**	−0.102 0.622	−0.064 0.756	1.000

Abbreviations: BAFF, B cell activating factor; BOP, bleeding on probing; OHS-I, Oral Hygiene Simplified Index (calculus index); PI, plaque index; *p* , *p* -value; *R* , Spearman correlation coefficient; TM, thalassemia major; TWEAK, tumor necrosis factor weak inducer of apoptosis.

a Correlation is significant at the 0.01 level (two-tailed). The significance level for the tests was set at *p* < 0.05. All boldfaced values indicate statistically significant results.

**Table 5:** Correlations of salivary and serum biomarkers and periodontal indices in the TM with healthy periodontium group (TMH)

		PI	BOP	OHS-I	Salivary BAFF	Salivary TWEAK	Serum BAFF	Serum TWEAK
PI	*R* *p*	1.000						
BOP	*R* *p*	**0.445** ** 0.026 a**	1.000					
OHS-I	*R* *p*	**0.466** ** 0.019 a**	0.175 0.403	1.000				
Salivary BAFF	*R* *p*	−0.009 0.965	0.207 0.321	0.023 0.915	1.000			
Salivary TWEAK	*R* *p*	0.046 0.828	0.150 0.474	−0.213 0.307	0.052 0.807	1.000		
Serum BAFF	*R* *p*	0.087 0.679	0.267 0.197	−0.133 0.527	0.018 0.930	0.058 0.784	1.000	
Serum TWEAK	*R* *p*	0.344 0.092	**0.583** **0.002**	0.126 0.548	0.370 0.069	**0.530** **0.006**	**0.494** **0.012**	1.000

Abbreviations: BAFF, B cell activating factor; BOP, bleeding on probing; OHS-I, oral hygiene simplified index (calculus index); *p* , *p* -value; PI, plaque index; *R* , Spearman correlation coefficient; TM, thalassemia major; TWEAK, tumor necrosis factor weak inducer of apoptosis.

a Correlation is significant at the 0.01 level (two-tailed). The significance level for the tests was set at *p* < 0.05. All boldfaced values indicate statistically significant results.

**Table 6:** Correlations of salivary and serum biomarkers and periodontal indices in the TM with healthy periodontium group (HG)

		PI	BOP	OHS-I	Salivary BAFF	Salivary TWEAK	Serum BAFF	Serum TWEAK
PI	*R* *p-*	1.000						
BOP	*R* *p*	**0.448** **0.025**	1.000					
OHS-I	*R* *p*	0.303 0.141	0.259 0.212	1.000				
Salivary BAFF	*R* *p*	−0.004 0.985	0.265 0.201	**−** 0.044 0.836	1.000			
Salivary TWEAK	*R* *p*	−0.016 0.939	**−0.535** **0.006**	−0.140 0.504	**−0.417** **0.038**	1.000		
Serum BAFF	*R* *p*	0.306 0.137	−0.093 0.658	0.025 0.907	−0.028 0.893	0.275 0.184	1.000	
Serum TWEAK	*R* *p*	**−0.402** **0.046**	−0.181 0.388	0.060 0.775	−0.007 0.974	−0.038 0.858	**−0.622** **0.001**	1.000

Abbreviations: BAFF, B cell activating factor; BOP, bleeding on probing; OHS-I, Oral Hygiene Simplified Index (calculus index); PI, plaque index; *p* , *p* -value; *R* , Spearman correlation coefficient; TM, thalassemia major; TWEAK, tumor necrosis factor weak inducer of apoptosis.

Note: Correlation is significant at the 0.01 level (two-tailed). The significance level for the tests was set at *p* < 0.05. All boldfaced values indicate statistically significant results.

**Table 7:** Correlations of salivary and serum biomarkers and periodontal indices in the control group (HH)

		PI	BOP	OHS-I	Salivary BAFF	Salivary TWEAK	Serum BAFF	Serum TWEAK
PI	*R* *p*	1.000						
BOP	*R* *p*	**0.712** **0.001**	1.000					
OHS-I	*R* *p*	0.038 0.857	0.150 0.476	1.000				
Salivary BAFF	*R* *p*	0.031 0.884	−0.004 0.985	0.088 0.676	1.000			
Salivary TWEAK	*R* *p*	0.099 0.639	0.185 0.376	0.053 0.802	**0.653** **0.001**	1.000		
Serum BAFF	*R* *p*	0.252 0.225	−0.031 0.882	0.123 0.559	0.189 0.365	0.142 0.497	1.000	
Serum TWEAK	*R* *p*	−0.066 0.753	0.202 0.334	−0.047 0.824	−0.391 0.053	**−0.423** **0.035**	**−0.580** **0.002**	1.000

Abbreviations: BAFF, B cell activating factor; BOP, bleeding on probing; OHS-I, Oral Hygiene Simplified Index (calculus index); PI, plaque index; *p* , *p* -value; R, Spearman correlation coefficient; TWEAK, tumor necrosis factor weak inducer of apoptosis.

a Correlation is significant at the 0.01 level (two-tailed). The significance level for the tests was set at *p* < 0.05. All boldfaced values indicate statistically significant results.

In the TMH group, serum TWEAK showed a positive, moderate correlation with BOP, serum BAFF, and saliva TWEAK ( *p* < 0.05) ( Table 5 ). In the HG group, as shown in Table 6 , a moderate negative association was found between PI and serum TWEAK, BOP and saliva TWEAK, as well as between salivary BAFF and salivary TWEAK ( *p* < 0.05). Serum BAFF and serum TWEAK displayed a strong negative association ( *p* < 0.001). In the HH group ( Table 7 ), there was a strong negative correlation between saliva BAFF and saliva TWEAK ( *p* < 0.001) and a moderately negative correlation between serum TWEAK and serum BAFF ( *p* = 0.002).

## Multiple Linear Regression Models

Multiple linear regression models were conducted to assess the relationship between biomarkers levels (salivary and serum BAFF and TWEAK) and the presence of TM and gingivitis. Age was used as a covariate in all models due to its statistically significant discrepancy across research groups. The models were designed to identify the effects of systemic (TM) and local (gingival inflammation) conditions on biomarker levels, while controlling for age as a potential confounding variable. Results were presented with *R* ^2^ , regression coefficients ( *β* ), 95% confidence intervals, and significance values ( *p <* 0.05). The detailed results regarding BAFF and TWEAK are presented in Table 8 .

**Table 8:** Multiple linear regression analysis

**Salivary BAFF** ***R*^2^ = 0.60 **					**Serum BAFF** ***R*^2^ = 0.77 **				
**Predictors**	**Coefficient B**	**95% CI lower**	**95% CI upper**	***p*** **-Value**	**Predictors**	**Coefficient B**	**95% CI lower**	**95% CI upper**	***p*** **-Value**
Thalassemia major	**215.02**	**101.31**	**328.73**	**0.0003**	Thalassemia major	**549.73**	**405.81**	**693.64**	**< 0.0001**
Gingival health status	**575.20**	**476.77**	**673.63**	**0.0001**	Gingival health status	**1009.15**	**884.58**	**1133.72**	**< 0.0001**
Age	7.53	−4.77	19.84	0.2274	Age	3.58	−12.00	19.15	0.6497
**Salivary TWEAK** ***R*^2^ = 0.76 **					**Serum TWEAK** ***R*^2^ = 0.65 **				
**Predictors**	**Coefficient B**	**95% CI lower**	**95% CI upper**	***p*** **-Value**	**Predictors**	**Coefficient B**	**95% CI lower**	**95% CI upper**	***p*** **-Value**
Thalassemia major	**38.64**	**23.69**	**53.60**	**< 0.0001**	Thalassemia major	**39.55**	**21.69**	**57.41**	**< 0.0001**
Gingival health status	**109.37**	**96.42**	**122.31**	**< 0.0001**	Gingival health status	**92.16**	**76.70**	**107.63**	**< 0.0001**
Age	0.03	−1.59	1.59	1.65	Age	1.22	−0.71	3.16	0.2125

Abbreviations: BAFF, B cell activating factor; CI, confidence interval; TWEAK, tumor necrosis factor weak inducer of apoptosis.

a *p* -value < 0.005 significant. The significance level for the tests was set at *p* < 0.05. All boldfaced values indicate statistically significant results.

## Discussion

In the current study, salivary and serum BAFF and TWEAK levels were significantly higher in both the TMG group as well as in systemically HG group, compared with the TMH and HH control group. Significant correlations were found between salivary and serum BAFF with salivary and serum TWEAK, suggesting that these biomarkers may be involved in the pathogenesis of TM and gingival inflammation. Multiple linear regression analysis demonstrated that TM and gingival inflammation were positively associated with elevated levels of salivary and serum BAFF and TWEAK.

The immune-inflammatory responses of the host play a pivotal role in shaping disease susceptibility. These complex biological processes influence the host's ability to defend against infections and its vulnerability to various health challenges. Understanding this interplay is essential for improving oral and systemic health outcomes. 29 30 The pathophysiology of periodontal disease is influenced by immune and inflammatory responses, which can exacerbate the systemic complications associated with TM. This study emphasizes the critical and often overlooked association between oral health and overall systemic health, where abnormalities of lymphocytes arising from systemic hematological disease in TM may potentially contribute to gingival inflammation. 5 31 Periodontal lesions are considered B cell lesions, given that biopsy specimens taken from affected periodontal tissues contain many B cells and plasma cells. Research has indicated that the activation, differentiation, and longevity of B cells and T cells are influenced by factors beyond antigens and T cell interactions, particularly cytokines from the TNF superfamily may play a role in this process. 32

BAFF and TWEAK have proinflammatory actions, participating in the innate immune response and affecting the transition to adaptive immunity. BAFF induces B cell proliferation and differentiation and is involved in the signal transmission between B cells and monocyte/macrophage cells. 10 There are three receptors associated with BAFF: BAFF-receptor (BR3), B cell maturation antigen (BCMA), and transmembrane activator, and calcium-modulator and cyclophilin ligand interactor (TACI) facilitate this interaction. The crosstalk between BAFF and these receptors activates the alternative nuclear factor kappa-B (NF-ҡB) signaling pathway and the classical pathway to a lesser extent, resulting in B cell survival and maturation. 33 Additionally, BAFF plays a role in T cell responses through its binding to BR3, which stimulates T cell activity; increased expression of BAFF has been shown to enhance the Th1 response. The levels of BAFF correlate with periodontal disease activity, as BAFF is found in saliva, serum, gingival tissue, and gingival crevicular fluid, with expression levels differing in individuals with periodontal diseases. 34

In association with its receptor fibroblast growth factor-inducible 14 (Fn14), TWEAK has been shown to induce prolonged NF-ҡB activation through both the classical and alternative pathways. Furthermore, TWEAK enhances the activation of the mitogen-activated protein kinase (MAPK) and protein kinase B (Akt) pathways. 35 Several studies on TWEAK in periodontal disease have identified its significant effect on immune and inflammatory responses, including the activation of proinflammatory cytokines such as interleukin (IL)-6, IL-8, matrix metalloproteinase (MMP)-1, and regulated activation, normal T cell expressed and secreted (RANTES), and angiogenesis. To the authors' knowledge, this is the first study that evaluates TWEAK levels in patients with TM and gingival inflammation. 36 TWEAK messenger ribonucleic acid has been frequently expressed in periodontally diseased tissue compared with healthy samples, indicating its role in modulating immune responses. 36 One immunomodulatory function of TWEAK is to prevent the potentially harmful overreaction of the innate immune response by supporting the deletion of activated NK cells upon immunological resolution. Additionally, TWEAK expression helps modulate the transition from innate to adaptive immunity in favor of Th1 response. 37 38

Alongside PI and BOP, utilized for classifying gingival inflammation according to established clinical criteria, the OHS-I (calculus index) was incorporated to offer a more comprehensive evaluation of oral hygiene status through the quantification of calculus levels. The purpose of this index was not to classify gingival status but to characterize overall hygiene, which may differ among groups and potentially affect gingival inflammation as a confounding variable. Furthermore, both PPD and CAL were assessed in the pilot study as well as in all study participants. It was determined that all TM patients exhibited no CAL and had a PPD ≤ 3 mm, which aligns with findings from previous studies. 21 39 40 The current findings demonstrate a significant correlation between salivary and serum BAFF and TWEAK with PI, BOP, and OHS-I, consistent with the previous studies on these cytokines in patients with various types of periodontal diseases. 17 34 36 It was shown that the concentration of TNF-α was significantly decreased, while IL-10 was significantly increased in BAFF blockade in mice with experimentally induced periodontitis. This result emphasizes the pathogenic contribution of BAFF to periodontal diseases by the potential therapeutic benefits of its neutralization. 41

Studies also revealed that patients with TM exhibit slight differences in oral hygiene compared with healthy individuals, with most of the research conducted on a younger age group (2–18 years), suggesting hormonal changes could affect gingival health during puberty. 18 19 The effect of iron overload in TM patients on the immune balance includes decreased phagocytosis and impaired T lymphocytes, which provide pathogenic microorganisms to initiate inflammatory and infectious diseases—a study by Fadel et al demonstrated that TM patients generally presented with moderately high levels of gingivitis, regardless of their iron overload level. 42 Gümüş et al reported that patients with TM had significantly greater levels of salivary MMP-8, MMP-9, tissue inhibitor of metalloproteinase-1 (TIMP-1), and serum TIMP-1 compared with systemically healthy participants. This finding suggested the possibility that TM could influence the inflammatory response. The patients had a greater PPD score and similar BOP and PI scores in the same study. In contrast, the present study showed greater PI and BOP scores in TM with gingivitis and in the systemically healthy with gingivitis groups. 39 Akcalı et al demonstrated elevated concentrations of salivary and serum APRIL, sRANKL, and IL-6, alongside an enhanced sRANKL/osteoprotegerin ratio and elevated levels of gingival crevicular fluid (GCF) sRANKL, IL-6, and IL-8 in comparison to systemically healthy participants. Additionally, it was found that BAFF is correlated with impaired activation and regulation of lymphocytes, which are associated with an autoimmune response in TM patients, potentially exacerbated by gingival inflammation. 40

BAFF and TWEAK, although not extensively researched in the context of gingivitis, have been examined in relation to periodontal disease linked with various systemic conditions, including diabetes mellitus, osteoporosis, and chronic migraine. Their inclusion in these settings points out their extensive immunomodulatory roles, especially in relating systemic immune changes with localized periodontal inflammation. The emphasis on BAFF and TWEAK arises from their roles in the TNF superfamily and B cell signaling pathways, which are important in the context of TM.

In the current study, patients with TM, characterized by systemic immune dysregulation, exhibited elevated levels of BAFF and TWEAK during the early and reversible inflammatory stage of periodontal disease. This suggests a potentially exaggerated or modified local immune response influenced by systemic factors. Although these findings cannot predict BAFF and TWEAK as definitive diagnostic markers for gingivitis, they provide insights into the potential influence of systemic conditions on local inflammatory pathways. Examining biomarkers in gingivitis cases offers insights into early pathogenic mechanisms and aids in identifying patients at risk for severe disease progression, particularly within medically compromised populations.

Saliva is readily available and easily collected without special equipment; additionally, it contains numerous components that are also present in the blood, facilitating the expansion of saliva-based diagnostics beyond oral disorders to encompass various systemic conditions. Saliva functions as an important biomarker for evaluating the body's physiological state, thus serving as a crucial resource for assessing both oral and systemic health. 43 44 The analysis of serum plays a crucial role in understanding the inflammatory stimuli and the corresponding reactions elicited by periodontal pathogens in the bloodstream. Research emphasizes the significance of serum in the study of periodontal diseases, revealing that individuals diagnosed with these disorders exhibit elevated levels of circulating cytokines. 45 The results of this research may provide valuable insights into the relationship between systemic health and oral conditions within this specific patient population. Poor oral hygiene practices and malocclusion in patients, exacerbated by their illness, immune dysregulation, and frequent hospitalizations, may negatively impact periodontal health and elevate the risk of gingival inflammation.

This study has several limitations that should be acknowledged. Although there was an age discrepancy between patients with TM and systemically healthy controls, multiple linear regression analysis indicated that age did not significantly influence the outcomes. Additionally, the study did not include concurrently measured, well-established inflammatory biomarkers such as IL-6, IL-8, TNF-α, and IL-1β. While our findings suggest a possible role for BAFF and TWEAK in the inflammatory profile of gingivitis in TM patients, further research incorporating a broader panel of inflammatory markers and formal diagnostic evaluations is necessary to enable more comprehensive and comparative profiling of the periodontal immune response in this patient population. The present study offers a cross-sectional assessment; however, longitudinal studies may yield more comprehensive insights into the response of these biomarkers to therapeutic interventions, such as professional dental cleaning and enhanced oral hygiene practices. Establishing a correlation between the alteration of biomarkers and clinical improvement would reinforce their significance in the inflammatory process and position them as objective tools for monitoring treatment outcomes in this patient population.

## Conclusion

This study demonstrated that salivary and serum levels of BAFF and TWEAK were significantly elevated in TM patients with gingival inflammation compared with healthy controls. These findings suggest a potential immunological interplay between systemic conditions like TM and early periodontal inflammation. Rather than establishing a direct causal link, the observed elevations in BAFF and TWEAK may reflect underlying systemic immune dysregulation in TM that influences the local periodontal environment. Given the known roles of these cytokines in immune modulation, their presence in gingival inflammation supports their possible utility as intermediary biomarkers in the systemic–oral health axis. However, the cross-sectional nature of the study and the absence of direct comparisons with classical periodontal markers (e.g., IL-1β, IL-6, TNF-α) limit the extent to which these results can be generalized.
